# Multiple myeloma: Detection of free monoclonal light chains by modified immunofixation electrophoresis with antisera against free light chains

**DOI:** 10.1016/j.plabm.2021.e00256

**Published:** 2021-10-12

**Authors:** Dorian Wilhite, Ahmed Arfa, Thomas Cotter, Natasha M. Savage, Roni J. Bollag, Gurmukh Singh

**Affiliations:** Department of Pathology, Medical College of Georgia at Augusta University, 1120 15th Street, BI 2008A, Augusta, GA, 30912, USA

**Keywords:** Multiple myeloma, Monoclonal light chains, MASS-FIX/MALDI, FLC-Modified SIFE, Serum free light chains, Light chain predominant multiple myeloma, Light chain myeloma, Minimal residual disease

## Abstract

**Introduction:**

Neoplastic monoclonal gammopathies are frequently associated with production of excess free monoclonal light chains. A sensitive method for detecting free monoclonal light chains in serum could provide a marker for residual/minimal residual disease and as an adjunct to serum protein electrophoresis to serve as a screening method for monoclonal gammopathies.

**Methods:**

Conventional serum immunofixation electrophoresis was modified by applying undiluted serum samples, and staining for serum free light chains with antisera specific to free light chains. Washing/blotting of gels was enhanced to remove residual proteins that did not bind to the antibodies including intact monoclonal immunoglobulins. Results from this modified immunofixation electrophoresis were compared with results from commercially available MASS-FIX/MALDI assay.

**Results:**

Monoclonal free kappa light chains could be detected to a concentration of about 1.78 mg/L and monoclonal free lambda light chains to a concentration of about 1.15 mg/L without the need for special equipment. Comparison of these thresholds with parallel results from a commercially available MASS-FIX/MALDI assay revealed the modified electrophoretic method to be more sensitive in the context of free monoclonal light chains.

**Conclusions:**

Modified serum immunofixation electrophoresis has been shown to detect low levels of monoclonal free light chains at a sensitivity suitable for the method to be used in detecting minimal residual disease, and potentially in a screening assay for monoclonal gammopathies. The disparity in the results with commercially available MASS-FIX/MALDI assay is postulated to be due to limited repertoire of reactivity of nanobodies of camelid origin.

## 1 Introduction

Neoplastic monoclonal gammopathies (NMG) consist of monoclonal gammopathy of undetermined significance (MGUS), asymptomatic or smoldering multiple myeloma (SMM) and multiple/plasma cell myeloma (MM) [[1], [2], [3]]. The diagnostic criteria for these entities are well described and generally accepted [4]. Of these only the malignant entity, MM, is treated in routine clinical care with antineoplastic drugs. By contrast the pre-malignant conditions of MGUS and SMM are usually observed and treatment initiated when the lesions meet the criteria for MM [[5], [6], [7]].

Multiple myeloma is a malignant tumor of plasma cells and is generally associated with synthesis and secretion of monoclonal immunoglobulins by tumor cells. MM is the commonest hematologic malignancy in adults [4]. The tumor is treatable but incurable. Improvements in drug treatment and autologous stem cell transplantation (ASCT) have improved survival such that survival beyond ten years is not uncommon [[8], [9], [10], [11]]. Staging systems for myeloma including the Durie-Salmon and International System take into account clinical and laboratory parameters of the extent of disease in addressing prognosis. Factors inherent to the tumor that portend poorer outcomes are the presence of del (17p) and/or translocation t(4;14), t(14;16), t(14;20), and amplification of 1q21. Partial or complete deletion of chromosome 13, 17p13 deletion and deletion 1p are additional markers of adverse outcome. The plasma cell labelling index (PCLI) may predict time to disease progression and death though currently PCLI is rarely used because of the availability of more practical prognostic methods [12].

Higher levels of serum free monoclonal light chains (SFLC) have been observed to result in shorter survival, perhaps through induction of renal damage. In MM lesions secreting intact immunoglobulins, a sub-group of about 18% of the tumors produce marked excess of free monoclonal light chains. These light-chain-predominant MM (LCPMM) have significantly lower eGFR and shorter survival. On analyzing SFLC and intact monoclonal immunoglobulin levels, the inflection point for identifying this subgroup of LCPMM was observed to be at 67 mg/L of SFLC per gram/dL of intact immunoglobulins for kappa light chain associated lesions. The corresponding value for lambda light chain associated lesions was 43.5 mg/L/g of monoclonal immunoglobulin [13].

About 15% of MM lesions secrete light chains only, i.e., light chain multiple myelomas (LCMM). Within this group of LCMM about 40% of the lesions have markedly higher levels of SFLC. The inflection point for separating high level of SFLC was observed to be at 455 mg/L. Due to the smaller number of patients observed separate inflection points for kappa and lambda lesions were not calculated. As in the case of LCPMM, the high SFLC subgroup of LCMM exhibited significantly lower eGFR and significantly shorter survival. No specific, effective treatments are available for addressing high monoclonal free light chains. Use of plasmapheresis and dialysis with a larger pore membrane have not shown consistent beneficial results [14].

Traditionally, gel or capillary based electrophoretic methods have been used for detection, quantification, and monitoring of monoclonal immunoglobulins. Gel-based methods employ serum protein electrophoresis (SPEP) and immunofixation protein electrophoresis (SIFE) and are standard laboratory tests at most medical centers. Capillary zonal electrophoresis (CE) and immunosubtraction electrophoresis (ISUB) are equivalent techniques using a more automated capillary fluidic electrophoresis method. The concentration of monoclonal immunoglobulins (MIg) in neoplastic monoclonal gammopathies is generally measured by densitometric scanning of monoclonal peaks on gel electrophoresis, or by the measured peak area guided by immunosubtraction (ISUB) on CE. Quantification by these two methods produce comparable results [15,16].

A major innovation has been the introduction of an assay for assessing free light chains in serum initially described by Bradwell et al. [17] Multiple assays are available for quantification of serum free light chains, though different methods do not produce comparable results [4]. Based on the molecular mechanisms driving immunoglobulin rearrangement during the synthesis of immunoglobulins, plasma cells tend to produce more light chains than heavy chains. This excess production of light chains extends to NMG. In neoplastic disorders of plasma cells, serum and urine contain free monoclonal light chains and these can serve as diagnostic and monitoring tools for NMG [[18], [19], [20]]. In LCMM and LCPMM, measuring and monitoring of SFLC provides a practical method for monitoring the course of disease [13,19]. The proposed role for enumerating the ratio of kappa to lambda SFLC has not proven to be particularly useful due to a high incidence of false positive, false negative, and incongruent results especially following autologous stem cell transplantation [4,[21], [22], [23]]. In general the NMG produce more kappa chains than lambda chains and this disparity complicates the results of involved/uninvolved light chain ratio especially following hematopoietic stem cell transplants [23].

A newer advance in detecting monoclonal immunoglobulins, in general, and monoclonal light chains, in particular, has been the use of mass-spectrometry. Nanobody-mediated concentration of immunoglobulins followed by matrix desorption time of flight analysis (MALDI-TOF) has been described as a screening tool for monoclonal immunoglobulins and presented as an assay with higher sensitivity than conventional methods. It has been promoted for detection of minimal residual disease (MRD) [[24], [25], [26], [27]]. However, at this time quantitation of monoclonal immunoglobulins is still reliant on densitometric scanning of SPEP or with CE, by using ISUB to guide demarcation. In principle mass spectrometry could be used to quantify monoclonal immunoglobulins or monoclonal SFLC.

Neoplastic gammopathies that predominantly (or exclusively) produce free light chains can be hard to detect, especially when looking for minimal residual disease in the post-treatment setting. The current standard of practice is overly reliant on the serum free light chain assay, which is fraught with false positives, false negatives, and an intrinsic inability to differentiate monoclonal from polyclonal light chains [4,13].

Standard IFEs frequently fail to detect monoclonal free light chain bands due to a number of factors, including (a) over-dilution of serum per standard protocols, (b) comigration of the monoclonal light chain bands and intact monoclonal immunoglobulins precludes distinction of light chains from the intact immunoglobulin band, (c) and possibly due to relatively poor binding affinity between conventional anti-kappa and anti-lambda antisera and monoclonal free light chains compared to standard antisera's affinity for light chains complexed to heavy chains.

A method using ultrafiltration to separate and concentrate SFLC followed by SIFE and densitometric scanning (QUIET) has been shown to have a detection limit of about 1.0 mg/L of monoclonal SFLCs [28]. A study to ascertain the nature of multiple bands on SIFE in a patient with LCPMM, provided the impetus to explore the use of polyclonal antisera to free light chains in SIFE for more sensitive detection of monoclonal SFLC in serum, i.e., detection of MRD, and this venture is the subject of this report [29].

## 2 Methods

This investigation was carried out at a 480 bed, tertiary care hospital, affiliated with a medical school in the Southeastern USA. The medical center offers matched unrelated donor, allogeneic, umbilical cord blood, and autologous stem cell transplants for hematologic malignancies along with providing other tertiary care and oncology services to the region. The study protocol was reviewed and approved by the Augusta University institutional review board (Protocol # 657783).

Specimens submitted for SPEP/SIFE were analyzed and results reported by the standard clinical methods as has been described previously [4,21,22]. Quantification of SFLC was conducted by using kits procured from The Binding Site and assayed with an Optilite analyzer. Rabbit polyclonal antisera to kappa and lambda free light chains were procured from SEBIA Inc. (Norcross, GA, USA.) Residual clinical serum samples were assessed for monoclonal SFLC by a Modified SIFE procedure, as described below: (The Modified SIFE will be designated FLC-Modified SIFE).

1. Undiluted serum samples were applied to SIFE gels procured from Helena, using Helena SPIFE touch equipment. (Helena Laboratories, Beaumont, TX).
  a. Some specimens were examined by triple application of the undiluted serum inoculum. This was done primarily to confirm negative results obtained via single application.
2. Electrophoresis was carried out according to the program and instructions provided the manufacturer.
3. Following electrophoresis, 50 μL of antisera to free kappa or lambda light chains were applied to selective electrophoretic slots and incubated according to the Helena protocol.
4. Following a standard incubation period with antiserum the antiserum was blotted with SIFE filters in Helena SIFE kits. Following blotting of excess antisera 50 μL of saline was added to the antibody slots and incubated for 3 min followed by blotting the excess solution as was done for the initial antiserum. This process was repeated two more times.
5. The gel was subjected to blotting with two filter papers, filter “C” and filter “D”, according to the manufacturer's protocol.
6. The gel was overlaid with fresh filter “C” and the filter paper was flooded with saline and incubated for 3 min. The filter paper was removed followed by blotting with filters “C” and “D”.
7. Application of filter C, flooding with saline and blotting with filters C and D was repeated twice more as per step 6.
8. The blotted gel was dried and stained according to the manufacturer's protocol.
9. The stained gel was evaluated visually.

**Fig. 1:**
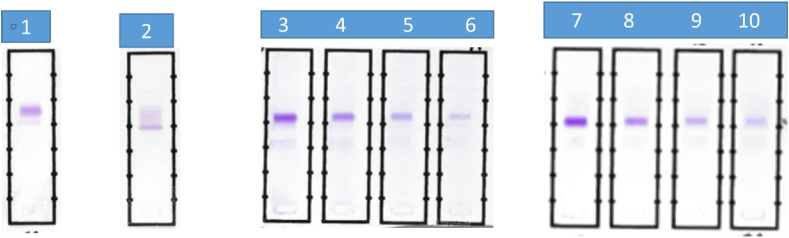
FLC-Modified SIFE with antisera to free kappa and lambda light chains. Lane 1 presents a typical band of monoclonal kappa light chains. Lane 2 represents a combination of polyclonal kappa light chains and a monoclonal band at the point of application. Lanes 3–6 are serial dilutions of serum from a patient with kappa MM treated with autologous stem cell transplantation. Patient's serum was serially diluted with pooled normal serum from 1:16 to 1:128 and monoclonal kappa light chain bands are detectable with decreasing intensity. Monoclonal kappa light chain was detectable (not shown) at a dilution of 1:256 but was not discernible at a dilution of 1:512 or higher. Lanes 7–10 are serial dilutions of serum from patient with IgG lambda MM. A separate lambda light chain monoclonal band was discernible on SIFE. Lanes 7–10 represent serial dilutions of serum from 1:4 through 1:32. A monoclonal lambda light band was detectable to a dilution of 1:256, representing a concentration of 1.15 mg/L for lambda SFLC.

**Fig. 2:**
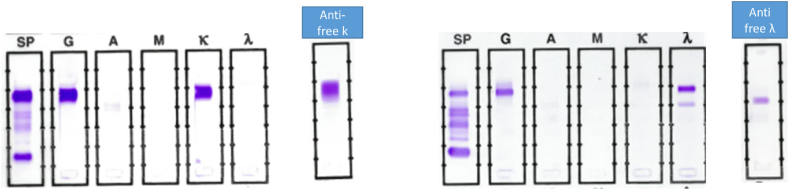
Representative gels from FLC-Modified SIFE are presented. The lanes marked SP, G. A, M, κ, λ, represent conventional serum immunofixation gels stained with appropriate antisera. The lanes marked anti-free k and anti-free λ represent FLC-Modified SIFE, stained with antisera to respective free light chains. In FLC-Modified SIFE, undiluted patient serum was applied and following staining with respective antisera, the gels were washed three times. The lane marked anti-free k shows free monoclonal light chains in the same location as the intact immunoglobulin (Left half of figure). Please note that in the lane stained with anti-free kappa undiluted serum was applied, whereas in conventional SIFE G and K lanes were at 1:10 dilution of serum. The broader band in the anti-free kappa lane represents the higher dose of inoculum. In the right half, conventional SIFE shows monoclonal IgG λ and free monoclonal λ light chains in a separate anodal band. Only the monoclonal λ light chain band is stained with the antiserum to free λ light chains.

Representative results from FLC-Modified SIFE are depicted in Fig. 1, Fig. 2.

Specificity of reactivity of the commercial antisera to free light chains was verified by staining sera from patients with known immunotypes of monoclonal gammopathies. For example in patient sera with a distinct kappa monoclonal light chain, separate from intact monoclonal immunoglobulin, the antiserum to free kappa chains was reactive with only the kappa light chain band and did not stain the intact immunoglobulin band. Similarly the antiserum to free lambda light chains did not stain known free kappa light chain monoclonal bands. The specificity was similarly confirmed for the reactivity of antiserum to free lambda light chains.

MASS-FIX/MALDI test results were purchased from a reference laboratory (Mayo Laboratories, Rochester MN). The specimens provided to Mayo Laboratories were not accompanied by any clinical information.

Specimens from selected patients with high concentrations of SFLC were subjected to serial dilutions with pooled normal serum from patients younger than 30-years of age. The patients selected for serial dilutions had diagnoses of MM with detectable monoclonal light chains by conventional SIFE. One patient each with kappa and lambda light chain myeloma and one patient each with IgG kappa or IgG lambda myeloma accompanied by readily detectable free monoclonal light chains by conventional SIFE were selected. The serial dilutions applied to the sera from these patients are noted in Table 1. A summary of parallel testing results is given in Table 2.

**Table 1:** Comparison of results by MASS-FIX/MALDI and FLC-Modified SIFE with Sebia antisera to serum free light chains.

No.	Diagnosis	Mass-Fix Result	Modified SIFE result	Kappa SFLC Conc.	Lambda SFLC Conc.	ASCT	DARA
	Monoclonal IgG kappa and free monoclonal kappa						
1	IgG K +K (1:32)^a^	IgG K	Kappa monoclonal	455.33/32 = 14.23	1.59/32	0	1
2	IgG K +K (1:64)^a^	IgG K	Kappa monoclonal	455.33/64 = 7.11	1.59/64	0	1
3	IgG K +K (1:128)^a^	IgG K	Kappa monoclonal	455.33/128 = 3.56	1.59/128	0	1
4	IgG K +K (1:256)^a^	Negative	Kappa monoclonal	455.33/256 = 1.78	1.59/256	0	1
	**Monoclonal IgG kappa**						
5	IgG kappa	IgG K	Polyclonal kappa	32.8	6.67	0	0
6	LCP IgG K	IgG K Elotuzumab/LC	Kappa monoclonal	15.97	0.44	0	0
7	IgG kappa	IgG K	Kappa monoclonal	10.7	1.53	0	0
8	IgG kappa	IgG K	Kappa monoclonal	10	1.25	0	0
9	IgG kappa	IgG K	Negative	29.48	0.28	0	0
	**Kappa MM**						
10	K MM (1:128)^b^	Negative	Kappa monoclonal	1224.23/128 = 9.56	2.04/128	1	1
11	K MM (1:256^b^)	Negative	Kappa monoclonal	1224.23/256 = 4.78	2.04/256	1	1
12	K MM (1:512)^b^	Negative	Negative	1224.23/512 = 2.39	2.04/412	1	1
13	K MM (1:1024)^b^	Negative	Negative	1224.23/1024 1.2	2.04/1024	1	1
14	K MM	Kappa	Kappa monoclonal	15.91	0.33	0	1
15	K MM	IgG K +K- Dara	Kappa mono + polyclonal	47.04	0.61	1	1
16	K MM	Negative	Polyclonal kappa	25.42	0.72	1	1
17	K MM	IgG K +K	Kappa monoclonal	16.21	0.59	0	0
18	K MM	IgG K +K	Kappa monoclonal	20.24	0.3	0	0
19	K MM	IgG K	Kappa monoclonal	17.79	0.2	0	0
20	K MM	Negative	Polyclonal K + POA mono	11.2	7.23	1	0
21	K MM	Lambda, IgG K, Dara	Polyclonal K+ POA mono	12.19	0.26	1	1
22	K MM	IgG K	Negative	2.81	0.37	0	0
	**Monoclonal IgG lambda and free monoclonal lambda**						
23	IgG L + L (1:32)^c^	IgG L +L	Lambda monoclonal	2.58/32	293.89/32 = 9.18	1	1
24	IgG L + L (1:64)^c^	IgG L +L	Lambda monoclonal	2.58/64	293.89/64 = 4.59	1	1
25	IgG L + L (1:128)^c^	IgG L+L	Lambda monoclonal	2.58/128	293.89/128 = 2.3	1	1
26	IgG L + L (1:256)^c^	IgG L + L	Lambda monoclonal	2.58/256	293.89/256 = 1.15	1	1
27	IgG L +L	IgG L	Lambda monoclonal	1.24	1.66	1	1
28	IgG L with h/o IgG L+L	IgG L +L	Negative	0.73	0.92	0	0
	**Monoclonal IgG or IgA lambda**						
29	LCP IgA Lambda	IgA L+ IgG K-Dara	Lambda monoclonal	0.41	19.41	0	1
30	IgG L MGUS	IgG L	Lambda monoclonal	2.08	5	0	0
31	IgG Lambda, +Alpha	IgG L+ IgA L	Lambda monoclonal	1.47	15.86	0	0
	**Lambda MM**						
32	Lambda MM (1:128)^d^	Lambda	Lambda monoclonal	1.28/128	1453.62/128 = 11.36	0	1
33	Lambda MM (1:256)^d^	Lambda	Lambda monoclonal	1.28/256	1453.62/256 = 5.68	0	1
34	Lambda MM (1:512)^d^	Lambda	Lambda monoclonal	1.28/512	1453.62/512 + 2.84	0	1
35	Lambda MM (1:1024)^d^	Negative	Lambda monoclonal	1.28/1024	1453.62/1024 = 1.42	0	1
36	L MM	Negative	Lambda monoclonal	10.63	14.21	0	1
37	L MM	cannot rule out mono	Lambda monoclonal	4.98	6.41	0	0
38	L MM	Oligo	Lambda mono + Polyclonal	11.93	18.28	0	0
39	Lambda restriction	IgG L	Lambda monoclonal	2.1	11.8	0	0
40	L MM	Oligo	Lambda monoclonal	8.49	15.17	1	0
41	L MM	IgG L	Lambda monoclonal	12.8	24.61	1	0
42	L MM	IgG K -Dara	Lambda monoclonal	6.61	3.56	0	1
	**Biclonal pattern**						
43	IgG K +IgG L	IgG K + IgG L	Kappa monoclonal	12.53	4.32	0	0
	**Non monoclonal**						
44	Polyclonal hyper gamma	Negative	Negative	21.71	11.19	NA	NA
45	Polyclonal; ESRD	Negative	Negative	55.9	19.62	NA	NA
46	Polyclonal- Sjogren	Negative	Negative	11.86	12.15	NA	NA
47	Pooled normal serum	Negative	Negative	8.52	6.63	NA	NA

Column labeled “diagnosis” provides the primary diagnosis of the monoclonal gammopathy. The superscripts a-d denote various dilutions from a single patient in each group. The dilution is indicated in parenthesis, and calculated concentration of involved SFLC is listed.

Lambda restriction in this column refers to a patient with a lambda monoclonal band on SIFE without a cognate heavy chain. The laboratory and clinical findings did not support a diagnosis of lambda MM.

The next two columns list the results from MASS-FIX/MALDI as provided by Mayo Laboratories and those obtained by FLC-Modified SIFE.

The columns Kappa and Lambda SFLC denote the results from SFLC assay. For specimens that were serially diluted, the raw SFLC concentration, dilution factor, and the estimated concentration of the involved monoclonal light chains are given, in order. The estimated concentration of monoclonal light chain is noted for only the involved light chain.

L = lambda light chain; K = kappa light chain.

ASCT – Autologous stem cell transplantation. 1 = ASCT done, 0 = ASCT not done.

DARA denotes patient treated with daratumumab. 1 = Dara administered; 0 = Dara not administered. No other therapeutic monoclonal antibody was used for the treatment of patients reported here.

POA refers to point of application. If a light chain monoclonal band was noted at the point of application, this observation was noted as a caution to not over-interpret an artefact at the point of application as a monoclonal band.

NA= Not applicable.

**Table 2:** Summary of results of comparison of MASS-FIX/MALDI and FLC-Modified SIFE for detection of monoclonal light chains.

No. of specimens with monoclonal Ig	43
Positive for monoclonal LC by both methods	18
Positive with FLC-Modified SIFE only	24
Positive with MASS-FIX/MALDI only	1

No. of specimens without monoclonal Ig	4
Negative by both methods	4

In addition to the parallel testing by MASS-FIX/MALDI, additional residual serum specimens from patients with (a) monoclonal gammopathies and (b) polyclonal increase in gamma globulins, without monoclonal gammopathy, were analyzed by FLC-Modified SIFE. The results depicting the detection of monoclonal light chains with reference to the total SFLC concentrations are presented in Table 3.

**Table 3:** Summary of results of all the specimens analyzed by FLC-Modified SIFE (including results in Table 1).

LC type, in NMG	N	Lowest Conc. Pos.	Highest Conc. Neg.
Kappa	87	1.78	29.48
Lambda	56	1.15	10.51

Polyclonal	19	NA	K = 128.18; L = 57.76

## 3 Results

FLC-Modified SIFE revealed monoclonal light chains in consonance with the expected findings, given a patient's diagnosis and immunoglobulin type determined by conventional SPEP and SIFE. Representative results from patients with light chain myeloma and intact immunoglobulin lesions with a separate band representing free monoclonal light chains noted by SIFE are shown in Fig. 1.

Serial dilutions of serum from patient “a”, in Table 1, revealed the limit of detection of kappa monoclonal light chains to be about 1.78 mg/L. This patient's primary diagnosis was IgG kappa MM and conventional SIFE revealed a separate band of monoclonal kappa light chains. MASS-FIX/MALDI detected monoclonal IgG kappa but did not identify free monoclonal kappa light chains. In patients with documented IgG kappa MM and kappa light chain MM, FLC-Modified SIFE displayed more results in consonance with the primary lesions. Serial dilutions of kappa LCMM, in patient “b”, identified a monoclonal band by FLC-Modified SIFE at a total SFLC concentration of 4.78 mg/L. MASS-FIX/MALDI did not detect monoclonal light chains at SFLC concentration of 9.56 mg/L, the highest concentration tested in this patient. On the other hand in some patients with kappa and lambda LCMM, MASS-FIX/MALDI identified intact monoclonal IgG kappa, or IgG lambda, when none was expected, based on the sum total of laboratory and clinical findings, e.g., specimen #s 15, 17–19, 21, 22, 39, 41, 42. In a one case each, monoclonal lambda was noted by MASS-FIX/MALDI in kappa MM and IgG kappa in lambda MM, specimen #21, 42 (Table 1).

Serial dilutions of serum from patient “c” revealed monoclonal lambda light chain by both FLC-Modified SIFE and MASS-FIX/MALDI at a concentration of about 1.15 mg/L. MASS-FIX/MALDI identified free lambda light chains in a patient with history of monoclonal IgG lambda and free monoclonal lambda light chains when FLC-Modified SIFE did not detect monoclonal lambda light chains. The total SFLC concentration of free lambda light chain was 0.92 mg/L in the specimen addressed above. Serial dilutions of serum from patient “d” with a diagnosis of lambda LCMM revealed monoclonal lambda light chains to a concentration of 1.42 mg/L by FLC-Modified SIFE while MASS-FIX/MALDI was positive to a concentration of 2.84 but not at 1.42 mg/L.

In a patient with a biclonal pattern of IgG kappa and IgG lambda, FLC-Modified SIFE detected monoclonal kappa light chains. Staining with anti-lambda antiserum did not detect free monoclonal lambda light chains. MASS-FIX/MALDI identified both the intact monoclonal immunoglobulins, i.e., IgG kappa and IgG lambda, but did not detect free monoclonal light chains of either type, specimen # 43.

As expected, in three patients with polyclonal increase in immunoglobulins and a specimen from pooled sera did not reveal a monoclonal light chain by either method.

The summary of comparative findings by FLC-Modified SIFE and MASS-FIX/MALDI are shown in Table 2 and demonstrate superior detection of monoclonal light chains by the FLC-Modified SIFE method. Summary of results of all specimens tested by FLC-Modified SIFE are shown in Table 3. Briefly, monoclonal kappa light chains were detected in specimens with total SFLC concentration as low as 1.78 mg/L for kappa and 1.15 mg/L for lambda. The highest levels of SFLC in patients with monoclonal gammopathy and negative results by FLC-Modified SIFE were 29.48 mg/L for kappa and 10.51 mg/L for lambda light chains. The highest levels of SFLC observed in patients, without detection of monoclonal light chains, were commonly seen in patients with end stage renal disease (ESRD). There was no evidence of monoclonal gammopathy in these patients. A similar phenomenon was also noted in patients with other conditions associated with polyclonal increase in gamma globulins, e.g., rheumatologic disorders and cirrhosis. The highest levels associated with negative results for monoclonal light chains, in patients without a diagnosis of monoclonal gammopathy, by FLC-Modified SIFE assay were 128.18 mg/L for kappa and 57.76 mg/L for lambda light chains.

## 4 Discussion

Progress in treatment of MM and the potential for a curative treatment with CAR-T therapy warrants improvement in methods for detecting minimal residual disease [30]. Mass Spectrometric analysis following nanobody mediated concentration of immunoglobulins (MASS-FIX/MALDI) has been described as a method for improved sensitivity and detection of MRD, though the results of MASS-FIX/MALDI were not compared with a reference methods or gas chromatography mass-spectrometry, or even urine examination. This method has also been promoted for use as a screening method for monoclonal gammopathies in lieu of SPEP and SIFE; and UPEP and UIFE, the current “gold standard”/reference method [[24], [25], [26], [27]].

Prompted by a prior investigation, we explored the use of polyclonal antiserum to free lambda light chains for detecting free monoclonal lambda light chains. To improve the sensitivity of the method, undiluted patient serum was applied to SIFE gels. It is noted that the manufacturer's protocol for SIFE entails 1:10 dilution of patient serum for staining with antisera to gamma heavy chains and kappa light chains and 1:5 dilutions for staining for mu, and alpha heavy chains and lambda light chains. The use of undiluted patient serum required additional wash steps for removing excess proteins not reacting with the antiserum. In preliminary studies it was noted that high concentrations of intact monoclonal immunoglobulins sometimes produced false positive result due to the monoclonal immunoglobulin not being washed out by the conventional SIFE protocol. The FLC-Modified SIFE procedure described here obviates such false positive results. In some patients with negative results on staining with antisera to free light chains, a triple application of the patient serum to SIFE gel was carried out to confirm the negative results.

While we tested for the specificity of reactivity of antisera to free light chains to the respective light chain, the possibility of a rare cross reactivity with other light chain could not be excluded, i.e. antiserum to free kappa light chain reacting with free lambda light chains. A more likely outcome is of a false negative result due to lack of reactivity of antiserum to free light chains of a give clonotype. This outcome was observed in one patient whose monoclonal light chain did not react with antiserum from one manufacturer, but did react with antiserum from a different manufacturer. The reverse was also observed in a different case.

In patients with lambda light chain associated lesions, either lambda chain MM or IgG lambda or IgA lambda MM, kappa free light chain concentration was sometimes higher than lambda SFLC concentration following treatment, especially ASCT. In one such patients with kappa dominant kappa/lambda ratio, for example, sample #42, detection of monoclonal lambda light chain and lack of detection of monoclonal kappa chain by FLC-Modified SIFE is likely to be due to high levels of kappa light chains being all polyclonal while all, or most, of the lambda light chains being monoclonal. The post-ASCT treatment abundance of polyclonal kappa light chains, including in patients with lambda light chain associated lesions, has been documented earlier [20,23].

The lack of detection of monoclonal light chains, by FLC-Modified SIFE, in patients with monoclonal gammopathy and SFLC concentrations above the detection limit of the method are postulated to be due to lack of sufficient amount of monoclonal light chains and the bulk of SFLC being polyclonal in nature, as is often observed in sera following treatment. For instance, in this context, it is likely that the bulk of kappa SFLC are polyclonal in sample #9; thus this would explain the lack of detection of monoclonal kappa light chains in this patient. Both polyclonal and monoclonal light chains were detected in some of the patients, as depicted in Table 1 and illustrated in Fig. 1.

To assess the relative sensitivity of the FLC-Modified SIFE procedure, samples were tested in parallel by MASS-FIX/MALDI at a reference laboratory (Mayo Laboratories, Rochester, MN). In this parallel evaluation de-identified specimens without clinical data were provided to the reference laboratory. As indicated in the results in Table 1, there was only one specimen in which MASS-FIX/MALDI detected a free monoclonal lambda light chain that was not detected by FLC-Modified SIFE. This patient had IgG lambda MM and had a history of monoclonal IgG lambda and free monoclonal lambda lights, earlier in the course of disease. In the sample noted as specimen #28, conventional SIFE was negative, and FLC-Modified SIFE did not detect monoclonal lambda light chains. Total lambda SFLC concentration in this patient was 0.92 mg/L and was apparently below the detection limit of FLC-Modified SIFE. In all other cases of disparate results between FLC-Modified SIFE and MASS-FIX/MALDI, the results with FLC-Modified SIFE displayed greater sensitivity for detection of monoclonal free light chains. In these instances the higher sensitivity for detection of monoclonal free light chains by FLC-Modified SIFE is bolstered by the results on serially diluted patient sera as documented in Table 1. Higher sensitivity by the FLC-Modified SIFE was always in consonance with the clinical findings and expected/predicted results. The FLC-Modified SIFE was not designed to test for intact monoclonal immunoglobulins and we are not able to comment on the results of MASS-FIX/MALDI showing monoclonal intact immunoglobulin when none was expected, as was seen in patients with light chain MM. However, detection of monoclonal intact immunoglobulins by MASS-FIX/MALDI in patients with LCMM raises the question of the validity of the method in detecting MRD. It is possible that intact monoclonal immunoglobulins detected in LCMM patients represent oligoclonal pattern in patients status-post chemotherapy and/or ASCT. To use the presence of monoclonal intact immunoglobulin in patients with MM would warrant proof that the monoclonal Ig detected by MASS-FIX/MALDI is identical to the original malignant clone. At this stage reporting of intact monoclonal immunoglobulins in patients with light chain myeloma could be considered a false positive result, casting doubt on the validity of MASS-FIX/MALDI detected monoclonal intact immunoglobulins indicating residual disease. This would be especially applicable to patients who had not undergone ASCT.

We postulate that the lower sensitivity of MASS-FIX/MALDI is a function of the limited repertoire of antibody activity in the camelid antisera/nanobodies used to enrich the pool of immunoglobulins. The nanobodies may not recognize certain unique epitopes in some monoclonal free light chains. This hypothesis is supported by our unpublished findings in a limited number of patient specimens tested in which the expected monoclonal free light chain was not detected by one reagent antiserum but was detectable by a second antiserum from a different vendor. It is likely that improved nanobodies with a broader repertoire of reactivity towards the unique epitopes in monoclonal light chains would improve the sensitivity of MASS-FIX/MALDI for expanded detection of free monoclonal light chains.

With respect to diagnostic algorithms for monoclonal gammopathy, it is conceivable that using usual SPEP, SIFE and FLC-Modified SIFE could detect all specimens with pathologic monoclonal immunoglobulins, including light chain only lesions. To maximize resource utilization, initial screening studies could be performed with SPEP and a single lane of undiluted patient serum stained with a mixture of anti-kappa and anti-lambda antisera. Serum specimens with a positive result by SPEP or FLC-Modified SIFE using mixtures of anti-kappa and anti-lambda antisera could be further analyzed by conventional SIFE, UPEP and UIFE.

While the data presented in this study suggest that the FLC-Modified SIFE method shows promise in improving detection of free monoclonal light chains, there are several potential caveats. Among the potential concerns regarding the use of the FLC-Modified SIFE assay are (a) increased labor to carry out multiple washes in addition to those in the conventional SIFE procedure, (b) cost of antisera to free light chains, and (c) modification in the reporting process for the electrophoresis algorithm. In this context, it may be appropriate to invoke this method only if and when detection of minimal residual disease becomes a clinically relevant issue. FLC-Modified SIFE would be used only if conventional SIFE and UIFE were negative for monoclonal light chains, or if urine was not available. The increase in technologist time amounts to about one additional hour per gel for the FLC-Modified SIFE procedure. Such an increase could be accommodated at most academic medical centers without the need to hire additional personnel. The increase in cost of reagents would be about $11.25 for one specimen and one antiserum type. This would be lower than the current cost of commercial MASS-FIX/MALDI at $145.00/specimen for a test with lower sensitivity. In-house testing would allow the cost to be recovered, once the test is approved by Centers for Medicare and Medicaid, through billing.

The salient findings in comparison with MASS-FIX/MALDI document frequent lack of detection of monoclonal free light chains by MASS-FIX/MALDI. In addition, MASS-FIX/MALDI detected intact monoclonal immunoglobulins in some patients with light chain only myeloma, even when the patient had not undergone ASCT. More concerning was the detection of monoclonal lambda light chains in specimen from a patient with Kappa MM. (Specimen #21). Monoclonal IgG kappa, in addition to DARA, was reported in a specimen from a patient with lambda MM who had not received ASCT, specimen # 42. We mention the lack of ASCT treatment as the latter can induce oligoclonal pattern that could be interpreted as monoclonal intact immunoglobulin. Also of concern was detection of Elotuzumab in specimen #6 when the patient had not received this therapeutic monoclonal antibody.

## Author statement

**Dorian Wilhite**: Data Curation, Methodology. **Ahmed Arfa**: Data Curation, Methodology. **Thomas Cotter**: Data Curation, Methodology. **Natasha M. Savage**: Investigation, Methodology, Project Administration, Writing Review and editing. **Roni J. Bollag**: Investigation, Methodology, Project Administration, Writing Review and editing. **Gurmukh Singh**: Conceptualization, Data Curation, Formal Analysis, Funding Acquisition, Investigation, Methodology, Project Administration, Resources, Software: NA, Supervision, Validation, Visualization: NA, Writing, Original draft, Writing Review and editing.

## Declaration of competing interest

Dr. Singh serves a consultant to Diazyme Inc. and HealthTap.
